# A Set of Time-and-Frequency-Localized Short-Duration Speech-Like Stimuli for Assessing Hearing-Aid Performance via Cortical Auditory-Evoked Potentials

**DOI:** 10.1177/2331216519885568

**Published:** 2019-12-20

**Authors:** Michael A. Stone, Anisa Visram, James M. Harte, Kevin J. Munro

**Affiliations:** 1 Manchester Centre for Audiology and Deafness, School of Health Sciences, University of Manchester, UK; 2 Manchester University Hospitals NHS Foundation Trust, UK; 3 Interacoustics Research Unit, c/o Technical University of Denmark, Lyngby, Denmark

**Keywords:** Cortical Auditory-Evoked Potential, auditory late response, modulations, hearing aids, auditory perception

## Abstract

Short-duration speech-like stimuli, for example, excised from running speech, can be used in the clinical setting to assess the integrity of the human auditory pathway at the level of the cortex. Modeling of the cochlear response to these stimuli demonstrated an imprecision in the location of the spectrotemporal energy, giving rise to uncertainty as to what and when of a stimulus caused any evoked electrophysiological response. This article reports the development and assessment of four short-duration, limited-bandwidth stimuli centered at low, mid, mid-high, and high frequencies, suitable for free-field delivery and, in addition, reproduction via hearing aids. The durations were determined by the British Society of Audiology recommended procedure for measuring Cortical Auditory-Evoked Potentials. The levels and bandwidths were chosen via a computational model to produce uniform cochlear excitation over a width exceeding that likely in a worst-case hearing-impaired listener. These parameters produce robustness against errors in insertion gains, and variation in frequency responses, due to transducer imperfections, room modes, and age-related variation in meatal resonances. The parameter choice predicts large spectral separation between adjacent stimuli on the cochlea. Analysis of the signals processed by examples of recent digital hearing aids mostly show similar levels of gain applied to each stimulus, independent of whether the stimulus was presented in isolation, bursts, continuous, or embedded in continuous speech. These stimuli seem to be suitable for measuring hearing-aided Cortical Auditory-Evoked Potentials and have the potential to be of benefit in the clinical setting.

## Introduction

Electric potentials can be recorded from the mammalian scalp in response to the presentation of acoustic signals. Due to the remoteness of the sites of generation from the sites of the electrodes, the potentials reflect the summation of neural activity generated in various stages in the auditory pathway, as the activity ascends from periphery to cortex (Burkard, Don, & Eggermont, 2006; Picton, 2011; Wunderlich & Cone-Wesson, 2006).

Evoked potentials can be used with relative ease in the clinic to establish estimates of auditory threshold in hard-to-test populations and hence can also be further used to prescribe hearing aid gains and verify subsequent audibility. The short-latency auditory brainstem response (ABR) has found much use in the clinic because it has a more reliable morphology than other responses and is unaffected by state of attention or arousal. However, ABRs, as their name suggests, do not provide evidence of a signal having ascended the full auditory pathway to the cortex. Alternatively, another low-latency response, the auditory steady-state response (ASSR) is generated from multiple loci along the auditory pathway. The influence of these higher loci, which do not include the cortex, can be mitigated by use of stimulus repetition rates of typically 80 to 90 Hz. With these high repetition rates, the low-pass nature of the ascending stages of the auditory pathway ensure that the overall response, like that of the ABR, is also dominated by generators in the brainstem.

The testing of activity higher up the auditory pathway requires measurement of the long-latency response. This response, with the longest delay relative to the presentation of the stimulus, mainly reflects activity in the primary and secondary cortex, the final destination of the evoked activity (other areas do also contribute, Stapells, 2002). Interest in this long-latency response, the Cortical Auditory-Evoked Potential (CAEP) as a clinical measure has varied over the years due to some disadvantages (Lightfoot & Kennedy, 2006; Wunderlich & Cone-Wesson, 2006), such as its morphology changing with age of the participant (Cone-Wesson & Wunderlich, 2003). Like the ABR and ASSR, CAEP responses are obligatory and so require no active response by the patient. Unlike the ABR and high-stimulus rate ASSR, the CAEP is modulated by the state of awareness of the participant. However, the CAEP does have several desirable properties for clinical applications:

1. It produces a large potential relative to the recording noise, hence short measuring time;
2. For short-duration stimuli (<100 ms), it is mostly produced by the onset of the stimulus (first 30 ms) (Picton, 2011; Wunderlich & Cone-Wesson, 2006), again contributing to clinically viable testing times;
3. The response reflects a change in the perceptible auditory world (Picton, 2011), indicative of an intact auditory pathway and, depending on stimulus, correlates with perception (Rance, Cone-Wesson, Wunderlich, & Dowell, 2002); and
4. Shorter duration signals (100 ms) produce larger CAEPs than longer duration (500 ms) (Agung, Purdy, McMahon, & Newall, 2006).

The CAEP is therefore a potential tool for verifying audibility in populations unable, or unwilling, to provide behavioral data (Hyde, 1997). Infants of developmental age less than 8 to 9 months form one candidate population since their poorly developed motor skills mean that they cannot give voluntary responses. For example, in England, hearing-impaired infants are on average fitted with a hearing aid by 82 days postpartum (Wood, Sutton, & Davis, 2015). This early diagnosis and remediation creates a need for verification of restoration of speech perception via the hearing aid. There have long been suggestions and reports of the use of CAEPs in the fitting of hearing prostheses (Cone-Wesson & Wunderlich, 2003; Korczak, Kurtzberg, & Stapells, 2005). Several reports in the literature used a short-duration speech-related stimulus as the acoustic stimulus for the measurement of CAEPs, to verify physiological detection of the stimuli, but not necessarily the validation of match-to-amplified targets. One rationale has been to use stimuli whose spectral distribution of energy show peaks at different frequencies, (Carter, Golding, Dillon, & Seymour, 2010; Pearce, Golding, & Dillon, 2007; Van Dun, Carter, & Dillon, 2012; Zhang et al., 2014). An alternative rationale for the use of speech-related stimuli is in the investigation of the ability to discriminate between speech features, for example frequency content (Agung et al., 2006), consonant–vowel transitions (Tremblay, Billings, Friesen, & Souza, 2006; Tremblay, Kalstein, Billings, & Souza, 2006) or voicing, place, and manner (Kuruvilla-Mathew, Purdy, & Welch, 2015), but those reports examined higher level speech-feature extraction rather than verification of hearing aid fitting, the latter being the original inspiration of this article. Speech appears to be a preferred stimulus for CAEP measures, because of its real-world applicability, but in comparisons between speech-tokens or tone-bursts as stimuli on a pediatric population, no particular preference was demonstrated in terms of efficacy of obtaining a response (Cone & Whittaker, 2013). More recent data by Bardy, Van Dun, and Dillon (2015) support use of stimuli broader in bandwidth than a pure tone to produce more reliable detections.

The HEARLab™ system (described in Munro, Purdy, Ahmed, Begum, & Dillon, 2011) is currently the only commercially available clinical test equipment for automated assessment of aided CAEPs and uses speech tokens for its stimuli. The stimuli are presented from a single calibrated loudspeaker sited in the free field in front of the participant. Stimuli are typically presented in blocks of 25 at the rate of 0.9/s, a rate used when collecting infant CAEPs using short-duration stimuli (e.g., Munro et al., 2011; Van Dun et al., 2012). A simple three-electrode montage is used for recording. Postprocessing of the recorded responses is used to generate an average waveform as well as a probability that a response was present. Typically about 80 to 100 presentations are necessary, producing a testing time similar to that required for short-latency responses, hence the attractiveness for clinical use. The use of an automated detection process, the Hotelling *T*^2^ test, removes the uncertainty in subjective determination of responses that would arise from the different morphology of the waveforms due either to age or participant (Carter et al., 2010). The stimuli supplied with the equipment have been excised from running speech and are labeled, /m/, /g/, /t/, and /s/, each token label reflecting the approximate spectral locus of the main energy peak of the particular stimuli. These stimuli have been postfiltered to reduce their spectral extent compared to their original production. In addition, the requirement for a short-duration stimulus, so as not to temporally smear the CAEP, means that these, as with other stimuli similarly reported, have been truncated in duration compared to those durations commonly encountered in conversational speech. We argue that such modified stimuli are “speech like,” but not necessarily speech. When compared to synthetic stimuli, their broader spectral extent as well as possible spectrotemporal contamination due to coarticulation effects, means that there is uncertainty as to the “what?” and the “when?” of the stimulus produced any evoked response.

In the context of a clinical measure of hearing aid fitting and performance in the acoustic free field, here we propose and assess the suitability of four new short-duration stimuli that are speech-like and are constrained in spectrotemporal extent. Bardy et al. (2015) showed that spectrally broader (one-octave), multitone stimuli produced a CAEP response detected more reliably than that elicited by pure tones in adults with normal-hearing. Hence the proposed two lower frequency stimuli are composed of multitone harmonic complexes. Since the two higher frequency stimuli overlap the frequency region where frication is dominant in speech, these two stimuli are comprised of inharmonic complexes, and hence are more noise-like. As all four stimuli are more frequency-specific than other speech tokens used in CAEP detection, we argue that they are better-suited for assessing the performance of the complete auditory pathway (from aid, via cochlea, and then neural transmission to the cortex) in targeted frequency ranges. They have also been designed to be robust against commonly encountered experimental deficiencies. In the remainder of the article, we report the design rationales that were used in the creation of the stimuli, report details of their computational generation, compare their free-field spectra and “erbograms” (a perceptual spectrogram) to those of excised real speech, and consider the effect of age-related changes in meatal length on the resulting cochlear excitation. After considering the statistical distribution of the levels of speech in different time windows and frequency bands to determine the necessary presentation levels, we provide some real-world validation by reporting two sets of proof-of-concept CAEP responses demonstrating that the stimuli perform as expected and finally assess the effects on the stimuli of the adaptive signal processing in four hearing aids.

## Design Rationales

The verification of hearing aid insertion gains, and hence audibility, in many brands of clinically based hearing-aid assessment equipment is performed using the International Speech Test Signal (ISTS; Holube, Fredelake, Vlaming, & Kollmeier, 2010), a recommended reference signal for measuring real ear responses and verifying hearing aid fittings (British Society of Audiology, 2018). Although other presentation levels can be used, a reference level of 65 dB SPL (a slightly lower level than “raised speech,” as defined by American National Standards Institute, 1997) is commonly used. Our overall goal was therefore to design narrowband stimuli suitable for the verification of prescribed insertion gains whose individual presentation levels would be the same as that measured in the same bandwidth of the ISTS long-term spectrum. For reasons to be described, their spectral shape does not follow that of the ISTS spectrum over their bandwidth. Therefore collectively, their spectra and relative levels are a stepwise approximation to the ISTS spectrum.

In addition to the stepwise spectral approximation, we set the following requirements:

1. The minimum frequency span of the stimuli should cover the bandwidth 400 to 4500 Hz, which contributes the bulk of the articulation, as modelled by the Speech Intelligibility Index (SII, see Table I of American National Standards Institute, 1997). This span is easily deliverable with modern hearing aids into the auditory meatus and verifiable using real-ear measurements. Three of the four signals lie within this frequency range. However, recent reports suggest that children with hearing impairment achieve multiple benefits from extending hearing aid bandwidth beyond 4 to 5 kHz (Brennan et al., 2014; Pittman, 2008; Stelmachowicz, Pittman, Hoover, Lewis, & Moeller, 2004). Very recent hearing aids demonstrate power bandwidths up to 10 kHz, so a fourth, high-frequency signal is included for purposes of future-proofing.
2. The frequency span should cover the same range over which a reasonable estimate of absolute threshold can be obtained by the ABR or ASSR, typically from above 500 to 8000 Hz. The bandwidth requirement is intended so that threshold estimates are comparable between the different techniques.
3. The stimuli should have a single onset and a single offset, each colocated in time across all frequency components contained within the stimuli.
4. The signals should not be so narrowband that their level is greatly modified by any of (a) a nonflat frequency response of the delivery transducer, (b) absorption by room modes (when using [pseudo-]free- or diffuse-field delivery), and (c) differences in meatal resonances due to the age of the participant. In addition, the bandwidth should be greater than the likely bandwidth of impaired (but functioning) auditory filters, typically a factor of three compared to normal widths (Moore, 1995).
5. The stimuli should produce a near-flat excitation pattern on the cochlea of a healthy auditory system so as to exercise the neural connections to a similar degree across the frequency span of the stimulus.
6. There needs to be confidence that any evoked response is produced from neural activity generated by cochlear regions close to the frequency span of the stimulus components. Therefore, the cochlear excitation of each stimulus should overlap only at a low level with adjacent stimuli. If there are errors in transducer amplification, or errors in estimate of auditory threshold, then the resulting unwanted spread of excitation will cause stimulation of an adjacent frequency region at a level insufficient, or unlikely, to be a major contributor to an evoked potential.
7. Synthetic stimuli can be crafted so that their onsets and offsets can be modulated (gated) to constrain the “spectral splatter” and consequently reduce the spectral extent of the neural activity of the cochlea contributing to the neural response. Some excised stimuli from real speech tokens used in CAEP testing have been observed to lack any gating.
8. In addition, the stimuli should take into account the recommended procedure produced by the British Society of Audiology for testing CAEPs (British Society of Audiology, 2016), which reflect current best practice in duration and rise times to reduce temporal smearing of the CAEP response. The short-duration requirement excludes the use of low rate (<100 Hz) modulation in the signal envelope. Higher rate modulations are acceptable and may be present due to intermodulation between tonal components.

## Generation of the Synthetic Stimuli

Alongside the theoretical design rationale detailed earlier, a practical guideline was to generate stimuli similar in frequency location to those supplied with HEARLab™ so as to build on recent experience of assessing audibility in an aided pediatric population (Van Dun et al., 2012). The spectral centers of energy for these stimuli are in a low-, mid-, mid-high-, and high-frequency band (additional design constraints, described later, mean that it is only practical to define four stimuli in the audio bandwidth of human hearing, further justification for referencing to the HEARLab choices). The loci of these energy centers approximate to the energy centres of /m/, /g/, /t/, and/s/, respectively. As will be shown later, real-world examples of the loci of these phonemes are not specific in frequency or time. Mirroring these phonemic descriptions, we designed the two lower frequency stimuli to comprise harmonic complexes, and so be tonal in nature, while the mid-high and high-frequency stimuli were comprised of a closely spaced inharmonic complex (16 components per auditory filter of a health adult, ERB_N_, Glasberg & Moore, 1990), so as to form (pseudo-) noise bands. The fundamental frequency of the harmonic stimuli was 140 Hz, nearly midway between that of adult male and female speech (106 and 170 Hz, respectively, Titze, 1989), but sufficiently low that even the low-frequency stimulus would comprise multiple harmonics within the stimulus bandwidth, reducing the effect of loudspeaker or room modes producing substantial departures from the intended presentation level. The period in digital samples of a single cycle at 140 Hz also has the advantage of being an integer, or small-integer-ratio divisor of the common audio sampling frequencies (32k, 44.1k, and 48k samples/s), hence the ability to make infinitely repeating sequences from short samples.

The initial design intended that each signal produced a mean target excitation level of 50 dB/ERB_N_, the level up to which healthy human cochlear filters do not appear to exhibit any variation of bandwidth with level (Glasberg & Moore, 1990). The spectral shape of the signal components was based on a uniformly exciting noise (UEN; Moore & Glasberg, 2000) whose spectrum produced equal excitation in each auditory filter of a healthy adult (ERB_N_), after correction for transmission from presentation in a diffuse acoustic field and passing through the healthy middle ear to the cochlea. The physical bandwidth used for each stimulus was either a minimum of two thirds of an octave or widened until it produced a cochlear excitation of a minimum of 4-ERB_N_. In loudness modeling, for impaired cochleae, auditory filters are assumed to reach a maximum broadening of 3.8-ERB_N_, by which stage the cochlear gain produced by the Outer Hair Cells is assumed to have disappeared (Moore & Glasberg, 2004). The excitation bandwidth therefore just exceeds the worst-case bandwidth of a single impaired auditory filter. An additional constraint was that the cross-over of adjacent excitation patterns was 30 dB less than the peak excitation, in order to ensure a large degree of spectral separation. For the low-frequency stimulus, the two-thirds octave bandwidth constraint would have meant the use of only two harmonics, otherwise the fundamental frequency, *f0*, would have to be reduced to unrealistically low values. A signal with only two harmonics would be more susceptible to level variations from loudspeaker imperfections and room modes as well as occupying only just over 3-ERB_N_ of cochlear bandwidth. A compromise was therefore necessary, so an extra harmonic was included, 280 Hz, at the lower edge of the band, and the lower edge of the range of frequencies amplified by the current generation of hearing aids.

**Figure 1.**
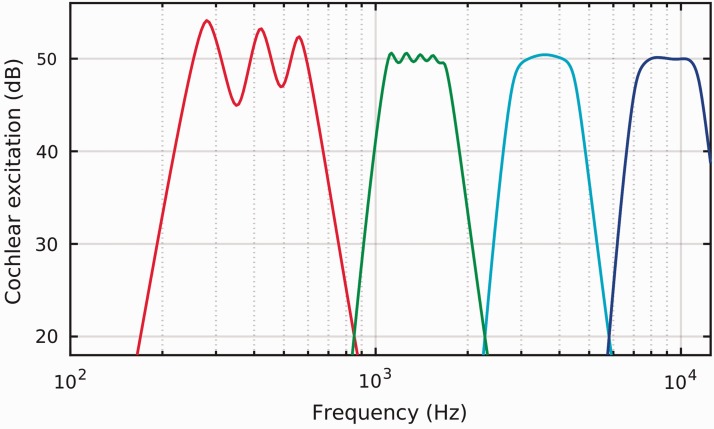
Excitation patterns as calculated for long-term versions of the stimuli, for a target excitation level of 50 dB. From left to right in the panel, the stimuli are the synthetic /m/, /g/, /t/, and /s/ (red, green, cyan, and blue, respectively).

The software “excit2005” (described in Moore, Glasberg, and Baer, 1997) was used to iteratively generate excitation patterns until the requirements for bandwidth and relative excitation level were met. Figure 1 shows the resulting patterns and represent the *ideal* estimated excitation of the cochlea due to the presence of a long-duration (several hundred ms) signal. Since the two lower frequency stimuli comprise harmonic tones, the peaks of the excitation patterns have a ripple, especially for the low-frequency signal. To calculate and compare excitation bandwidths across all stimuli, UEN bands were used to generate excitation patterns with the same width at the –3 dB points as for the harmonic versions.

The design parameters for the stimuli are given in Table 1, with the bandwidth comparison of the physical, noise-band equivalent UEN given in Hz, and the excitation spread in octaves and units of ERB_N._ The expression of the physical stimulus bandwidth as a noise band permits equating the stimulus level to the band power found in an average speech spectrum such as the long-term average speech spectrum (LTASS, Byrne et al., 1994; Moore, Stone, Füllgrabe, Glasberg, & Puria, 2008). Hearing aid test equipment is more commonly supplied with the female-talker ISTS signal (Holube et al., 2010), whose LTASS is matched to the LTASS of Byrne et al. (1994). The relative bandpowers have been calculated relative to this reference spectrum and are given in the final line of Table 1. To enable independent synthesis of these signals, the component frequencies and relative component levels are detailed in Table 1 of the Supplementary Material.

**Table 1.** Bandwidths of the Proposed Signals as a Function of Signal Parameters.

Stimulus band	Low	Mid	Mid-High	High
Harmonic numbers @ *f0*=140 Hz	2–4	8–13	NA	NA
UEN-equivalent bandwidth (Hz)	240–611	1,084–1,717	2,828–4,468	7,141–11,362
UEN-excitation –3 dB bandwidth				
Octaves	1.35	0.72	0.69	0.70
ERB_N_	5.5	4.0	4.2	4.4
Relative band power of stimulus compared tofull bandwidth, ISTS spectrum (dB)	–2.7	–14.5	–20.6	–21.9

*Note*. The UEN-equivalent bandwidth is the bandwidth of the rectangularly windowed UEN spectrum that produces the same excitation as the harmonic stimuli, measured at the –3 dB points. The final line gives the relative bandpower of the ISTS spectrum contained within the UEN-equivalent bandwidth. For a 65 dB SPL ISTS signal, the band powers would consequently be 62.3, 50.5, 44.4, and 43.1 dB SPL for the low-, mid-, mid-high, and high-frequency stimuli, respectively. The component frequencies and relative component levels are detailed in Table 1 of the Supplementary Material. NA = not applicable; UEN = uniformly exciting noise; ISTS = International Speech Test Signal.

At first sight, for a reference speech level of 65 dB SPL, the relative bandpowers are very low for the mid-high and high-frequency stimuli, around 40 to 45 dB SPL. These levels represent a part of the speech dynamic range that, for speech presented at 65 dB SPL, would be expected to be amplified to audibility through a well-fitted hearing aid, at least for a mild to moderate degree of loss (Keidser, Dillon, Flax, Ching, & Brewer, 2011; Moore, Glasberg, & Stone, 2010; Seewald, Moodie, Scollie, & Bagatto, 2005). The need for a possible refinement of choice of presentation level is discussed in a later section.

## Spectrotemporal Comparisons of Short-Duration Speech-Like and Synthetic Stimuli

The input to the excitation pattern software operates from spectral power densities and so makes no assumption about the duration of the signal. CAEP signals are commonly of short duration. Consequently, the onsets and offsets of the stimuli will generate modulation and widen the resulting excitation from the ideal. To make comparisons between speech-like CAEP stimuli and the new stimuli, short-duration versions of the new stimuli were generated, given cosine-squared ramps at onset and offset, and analyzed for their spectrotemporal content. Following the British Society of Audiology (2016) guidelines, the rise time, and half-amplitude-duration times, of the pip versions of the stimuli were, 20 and 80 ms for the low-frequency signal, and 10 and 70 ms for the remaining three signals. This equates to the same duration (60 ms) of the steady-state portion for each signal, but a proportionately longer rise time for the low-frequency signal in order to maintain a perceptually narrow bandwidth of “spectral splatter” due to the stimulus onset and offset.

We assembled three sets of short-duration real speech stimuli, alongside the new stimuli, to make a total of four sets. The first set comprised examples of speech tokens excised from running female speech, adjusted in duration and spectral content to avoid gross intrusion of adjacent vowels, as used in the HEARLab system. A second set was the synthetic stimuli described earlier.

The final sets were generated by excising speech tokens from two different corpora of speech recordings: one being running male speech recorded for the analysis contained in Moore et al. (2008) and the other being a male speaker of British English pronouncing examples of vowel-consonant-vowels (VCV), where the vowel (V) was /a/.

The durations of the first set were not adjusted for this analysis since they came from the HEARLab CAEP test set. The sets generated by excision were chosen to provide some variety from the HEARLab set in both speaker type and speaking style, and involved locating and waveform editing to extract consonants with the same phonemic label as the HEARLab stimuli. These last two sets were constructed with the durations and rise times outlined earlier for the new stimuli. Consequently, even for well-articulated consonants in the /a/C/a/ context, the stimulus duration was sometimes too long to capture just the consonant, so some leakage from the surrounding vowel occurred.

**Figure 2.**
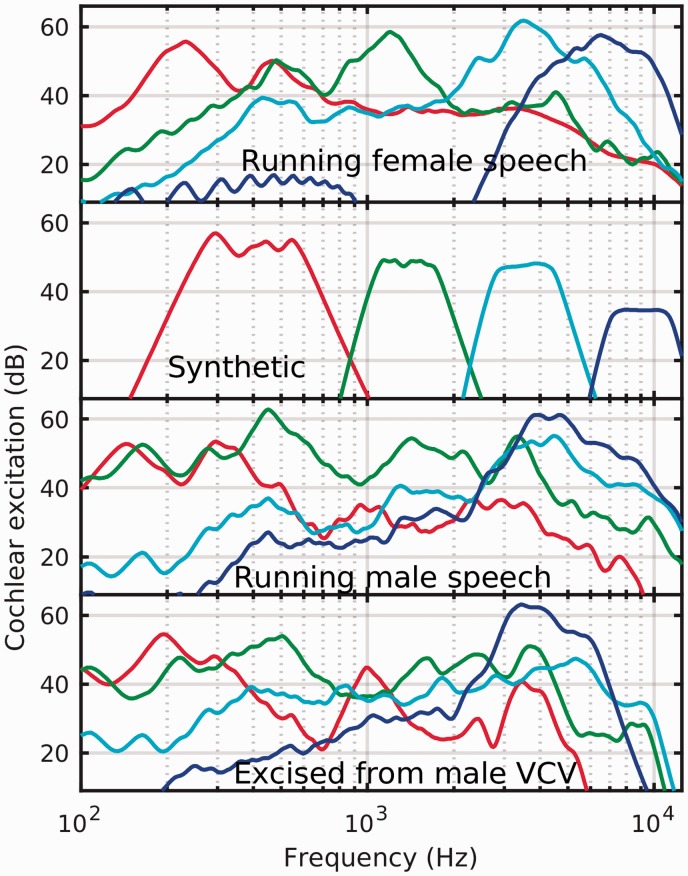
Cochlear excitation patterns averaged over each stimulus duration, for the low- (/m/, red line), mid- (/g/, green line), mid-high (/t/, cyan line), and high- (/s/, blue line) frequency stimuli compared as a function of stimulus source. The bottom row contains those stimuli excised from male VCV, the second row up contains those excised from male running speech, the third row up contains those excised from the synthetic stimuli, and the topmost row contains the tokens excised from female running speech. Within each panel, the level of the low-frequency stimulus was 65 dB SPL, and the remaining three stimuli are plotted at their intended presentation level relative to the low-frequency signal.

Figure 2 shows the resulting excitation patterns for the different stimulus sources, but separated to one source per panel. For each panel, the low-frequency stimulus from each set (plotted in red) was normalized to 65 dB SPL, and the other three stimuli from the same set analyzed with the same relative levels, otherwise unadjusted from the original recordings. The running female speech shows increases in the peak level with frequency of the separate stimuli. The male speech tends to show either flatter, or decreasing, level with increasing frequency. Disturbingly, from the perspective of using speech tokens for frequency-specific CAEP testing, there are several cases where, within a single stimulus, there is no distinct peak that is more prominent in frequency than any other. This is especially noticeable in the set produced from running male speech, but also seen with those from the male VCV stimuli.

**Figure 3.**
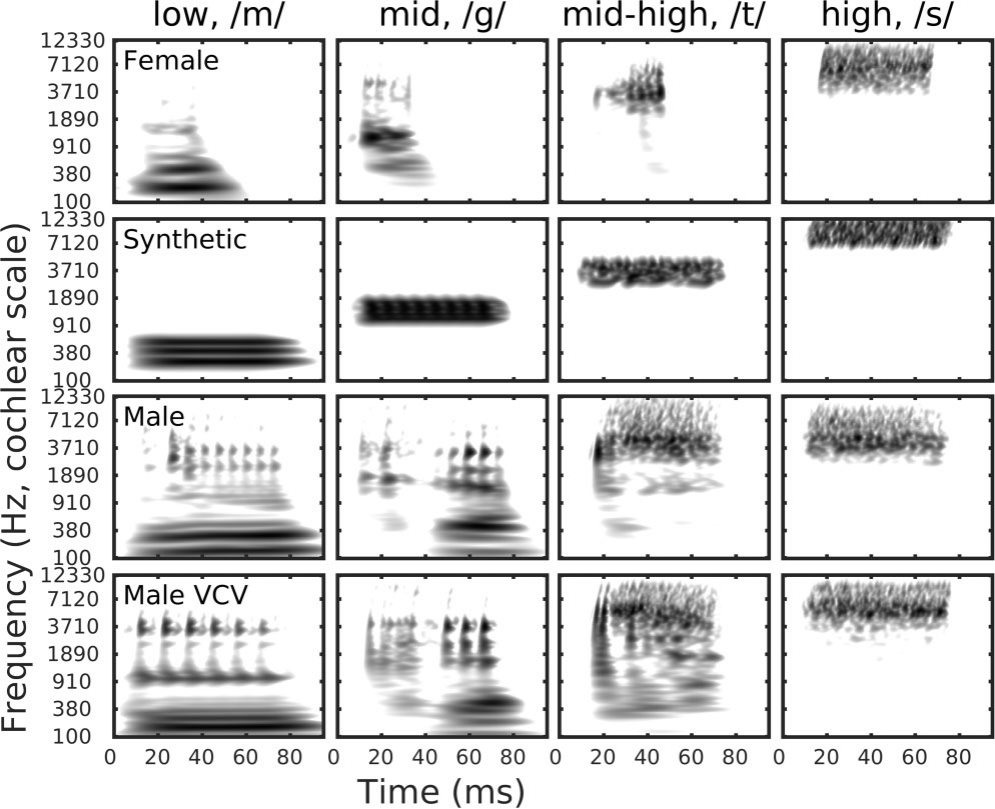
Erbogram representations of cochlear activity as a function of time, in columns from left to right for the low-, mid-, mid-high, and high-frequency stimuli compared as a function of stimulus source. The ordering of the stimuli source by row is the same as for Figure 2. The grayscale is normalized for each panel to cover a range of 30 dB, from black (most intense) to white (least intense). The ordering of the stimuli source by row is the same as for Figures 2 and 3.

Figure 3 shows the erbograms of the stimuli, on a time–frequency scale. For these plots, the darker the shading, the greater is the activity. An erbogram is similar in construct to a spectrogram, but the frequency analysis is performed by first taking into account the transfer in sound pressure from the free field to the cochlea, followed by frequency analysis performed by a level-independent auditory filterbank using fourth-order gammatone filters (Patterson et al., 1992). The erbogram therefore shows the evolution of cochlear excitation over time in response to a stimulus. The resulting patterns are consequently more indicative of the perceptual relevance of a signal than those produced by a spectrogram. In each subplot of Figure 3, the grayscale has been normalized so that the least intense level (white), is reached when the signal is more than 30 dB below the peak level (black). Each column compares a different stimulus, as labeled at the top of the column. From bottom to top, each row represents stimuli from male VCV, male running speech, the synthetic stimuli, and the female running speech.

Even ignoring the pitch-period modulations, there are several stimuli where there is a secondary onset partway through, and possibly occurring in a different frequency region, for example, low frequency for both female and male running speech, mid frequency for male running speech, and male-produced VCV. The spectral-excitation only plots of Figures 1 and 2 only show the temporal integration of the power throughout the duration of the stimulus. They do not distinguish between long-duration constant level features and short duration intense features occurring at any time during the stimulus. The peak level of these shorter duration secondary onsets, relative to the primary onsets, is therefore underestimated when viewed with no temporal axis. Since the CAEP for short stimuli represents a response to the onset of a stimulus (Picton, 2011; Wunderlich & Cone-Wesson, 2006), the presence of multiple onsets could produce an ambiguity as to which high-energy locus was responsible for triggering a detected CAEP.

## Effects of Age-Related Changes in Meatal Resonance

As the infant pinna and meatus grow, the acoustics, and hence resonances (and anti-resonances) move in frequency. Keefe, Bulen, Campbell, and Burns (1994) measured the transfer function of a signal from a diffuse field to a probe microphone in the meatus of infants as a function of age, primarily 1, 3, 6, 12, and 24 months. By 24 months, the pinna and meatal sizes were still not that of a fully grown adult, although the bulk of the variation had been achieved. At least for age 1 to 12 months, the majority of the variation was the downward drift in frequency of a double resonance starting around 4.5 and 5.5 kHz, and ending up around 2.8 and 4.5 kHz, close to that apparent in the same transfer function for adults specified in American National Standards Institute (2007).

**Figure 4.**
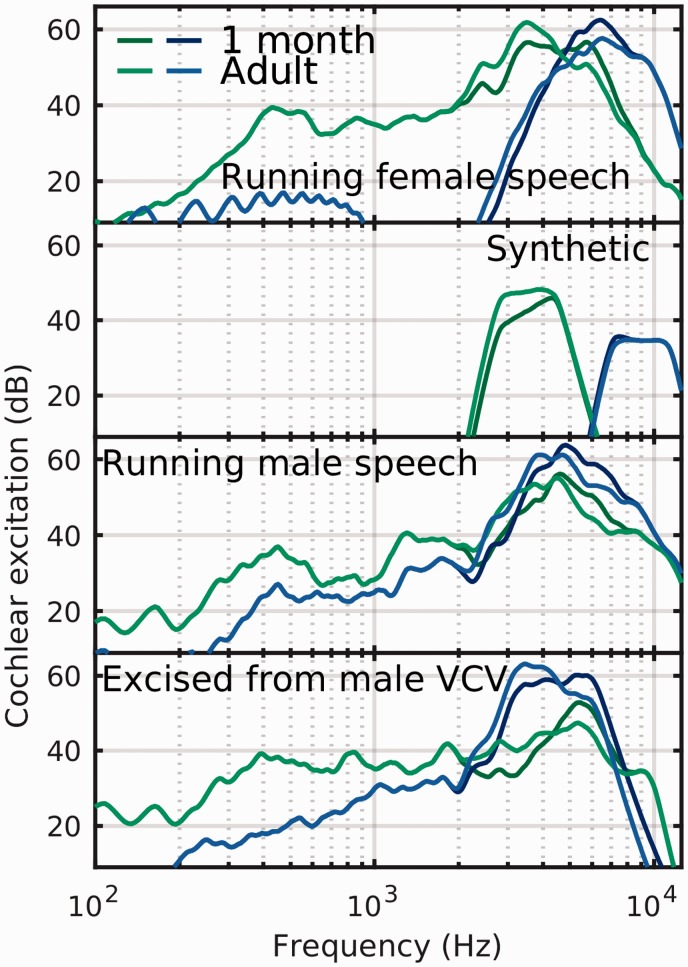
Similar to Figure 2, cochlear excitation patterns averaged over each stimulus duration, for the two higher frequency stimuli compared as a function of age, and hence average size of concha and meatus. Lighter colored lines are for adults and darker colored lines for 1-month-old infants.

Table II of Keefe et al. (1994) reported the one-third octave bands in which there was a significant change in meatal response with age. The majority of the changes occurred in bands centered on 2 kHz and above. Although lower frequency sections also change with age, the variation was not so drastic. Using the figures given in Figure 7 of Keefe et al. (1994), the standard adult diffuse field correction used in the excit2005 software (Moore et al., 1997) was reduced in level by the response of the double resonance of the 24-month-old and replaced with that of the double resonance of a 1-month-old. This approximates the maximum change likely to be seen in the transfer function with age, for frequencies exceeding 2 kHz. For the synthetic stimuli reported here, this is only likely to affect our mid-high and high-frequency stimuli. For purposes of comparison, the 1-month and adult-aged excitation pattern responses are plotted in Figure 4. The main changes in the patterns for the 1-month-old are the reduced level between 2 and 4.5 kHz, with an increase for components at frequencies exceeding about 4.5 kHz. For the broader band, speech-originated stimuli, the excitation peak moves upward in frequency. For the synthetic stimuli, although there is a reduction in overall stimulation, the center of gravity remains in-band to that of the adult response. The greatest reductions occur in the 2.5 to 3.5 kHz region. The mid-high frequency stimulus from running male and female speech appears to suffer the most drastic change since the excitation undergoes a near 1-octave shift (from 2–3 kHz to 5–6 kHz), leading to increased risk of a response from a spurious peak.

Overall, even for the most extreme change in meatal shape with age (from 1 month to adult), the changes in cochlear excitation are only seen in the two highest frequency stimuli. For the speech-like stimuli with a broad bandwidth, the potential exists for these changes to alter the location of the spectral peak, reducing the confidence in the *what* and the *when* of the stimulus produced any observed cortical response.

## Choice of Presentation Levels Across Stimuli for Validation of Hearing Aid Fitting

The common prescription formulae for hearing aids specify a gain as a function of frequency that is to be achieved when presented with a speech or speech-spectrum signal at a reference level, typically 50, 65, or 80 dB SPL. The last line of Table 1 references the necessary free-field relative presentation levels of the synthetic stimuli so that they have the same power as the mean power of the relevant bandwidth in a full bandwidth ISTS spectrum. These relative levels, declining with increasing frequency, greatly differ from the levels used for delivery of the equivalent stimuli by the HEARLab system. The presentation levels of the stimuli in HEARLab are measured using an impulse-weighted filter (I-weighting, incorporating a 35-ms time constant) and are set to the same level as for the mean level of the running speech from which the token was excised. For all except the low-frequency synthetic stimulus, the differences between the synthetic and the HEARLab stimuli therefore exceed 14 dB. Possible explanations for this difference could be due to either the difference in measurement used between HEARLab (I-weighting) and our signals (root mean square [RMS] of the full-power, i.e., nonramped, portion) or the duration (30–50 ms in HEARLab and 60–70 ms in our stimuli).

Since speech is a “peaky” signal (large crest factor), its variation is not properly captured by the specification of a mean spectrum. A more detailed analysis of the statistical variation of speech levels at two timescales, 10- and 125-ms duration windows, was reported in Moore et al. (2008). Briefly, they bandpass filtered excerpts of narrative speech into 2-ERB_N_ widths and generated cumulative histograms of the RMS level in overlapping windows of predetermined duration. The cumulative histograms were then plotted across frequency at pre-decided contours of interest, such as at 80%, 50%, 20%, 10%, 5%, 2%, and 1%. These contours were labeled “Exceedances” since they defined the rate of occurrence, relative to the mean level, for which the level in a particular window duration exceeded that contour. Independent of the two timescales, 125 and 10 ms, the mean level of a speech signal was determined by approximately 10% to 20% of the measurement timeframes, that is, a relatively modest frequency of occurrence.

Here, the interest is in the discrepant level difference between the HEARLab stimuli and the proposed stimuli. Are the higher relative levels of the HEARLab stimuli representative of real speech? Since the relative levels of the HEARLab /g/, /t/, and /s/ signals were higher than the 1-% exceedance levels previously reported, exceedance values were recalculated to ignore the higher exceedance percentages and concentrate on the lower percentages, especially below 1%. To obtain a more reliable estimate of the sub -1% levels, the data set on which the Moore et al. (2008) figures were generated was expanded using additional recordings to increase the total number of talkers to 18 (10 males and 8 females, previously 6 and 8, respectively), and reanalyzed for a narrower range of exceedance levels from previously. The additional recordings were available from a data set recorded under very similar conditions to those used in Moore et al. (2008). Collectively, the recordings represent in excess of 1,000 s of narrative speech. To address a possible reason for the difference in level measurements between the two sets of stimuli arising due to the timescales of the level measurements, a shorter time window for calculating exceedances than used previously was also included.

**Figure 5.**
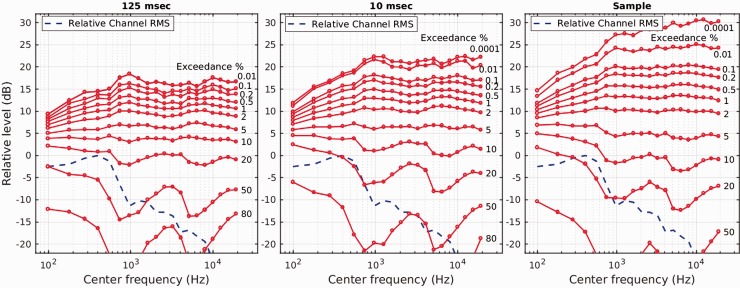
Exceedances for speech prose, as described in Moore et al. (2008), generated at three timescales, 125-ms (left panel), 10-ms (middle panel) and sample duration (at 44.1 kHz, right panel), and for very low exceedance rates. Within each 2-ERB_n_-wide channel spanning the audio frequency range, the levels within in a predetermined time window are measured and formed into a histogram as a function of level. Each red line shows the level relative to channel RMS for which the signal in a channel exceeds a certain percentage of the time windows. The data represent the cumulative statistics of over 1,000 s of narrative speech. See text for further details.

Exceedances calculated at three different timescales and including sub -1% levels are shown in Figure 5. Durations of 125 and 10 ms, as previously, are shown in the left-hand and middle panels, but additionally, at sample duration (for a sampling rate of 44.1 kHz) in the right-hand panel. So as to provide greater clarity at the very low exceedance rates, the data were averaged across both male and female talkers. Of interest across all three panels is that, for exceedance rates between 1% and 5%, the level is remarkably constant both across frequency and window duration, for example, for 1% exceedance, at around 11 to 13 dB relative to channel mean. It is only for exceedances below 1% that a marked variation with window duration starts to become apparent; even then it is only around 4 dB different at 0.01% for 125 and 10 ms duration windows. It is primarily the sample-duration window that shows a much greater difference from the other two window durations at these very low exceedance rates.

Irrespective of window duration and possible confound with measurement method (impulse or RMS), levels 14 to 20 dB above mean level (the 0-dB line in each panel) occur only relatively infrequently, less than 0.5% of the time. Eliciting a cortical response with a stimulus level that occurs this infrequently in running speech therefore does not necessarily validate the audibility of a range of speech levels that is typically required to obtaining good representation of the articulations (American National Standards Institute, 1997).

We propose that the intended presentation levels for the new stimuli should be the same level as the bandpower from the ISTS signal at the reference level used for the hearing aid gain prescription since they are more representative of the statistical distribution of levels found in speech. Differences in analysis window duration do not appear to be the reason for the difference between HEARLab presentation levels and those for our stimuli. In addition, analysis of the speech excerpts show that narrowband signals rarely achieve anywhere near the mean full-bandwidth speech level except either at a very low frequency of occurrence, or at audio frequencies occupied by low-frequency test stimulus.

However, for more severe losses, it is common for either the gain prescription algorithm, or the hearing aid wearer, to request the gain to be reduced (Keidser, Dillon, Carter, & O’Brien, 2012; Moore, 2012), especially at high frequencies in the case of typical presbyacusic losses. Therefore, the theoretical presentation levels detailed in Table 1 may be insufficient if the prescription algorithm does not intend to amplify the mean band level to audibility, other than at very high speech levels.

An additional factor for determining the required presentation level is that in order to achieve an 80-% probability of detection of a CAEP response, (pure-tone) signals need to be presented at about 6.5 dB above absolute threshold (Lightfoot & Kennedy, 2006).

In summary, the use of CAEPs in a clinical setting to verify audibility via hearing aids may therefore need to refine the theoretical presentation levels based on the minimum level expected to elicit a response. This minimum level is a complex mix of speech statistics, hearing aid prescription formulae, subjectively driven fine tuning, stimulus content, and detection statistics. Clinical use of CAEPs seems likely to require greater integration between the fitting software and CAEP measurement equipment so as to be better able to interpret the significance of any elicited response.

## CAEP Responses From Adults Using Either the HEARLab or the Proposed Stimuli

Recordings of evoked responses were performed on two adults in response to free-field binaural presentation of either the HEARLab /m/, /g/, and /t/ stimuli or the proposed low, mid, and mid-high stimuli. Full details of the presentation method are given in the Supplementary Material.

**Figure 6.**
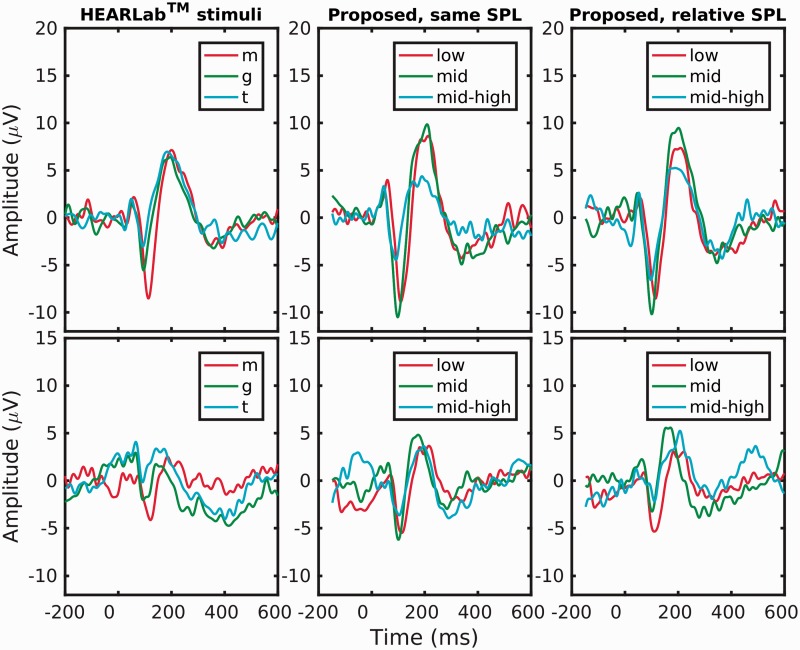
Comparison of EEG recordings taken from either a middle-age male (top row) or a young female (bottom row). The left-hand panel shows responses to the HEARLab stimuli for a presentation level of 65 dB SPL. The middle panel shows responses to the three lower frequency proposed stimuli, again for a presentation level of 65 dB SPL for each stimulus. The right-hand panel shows responses to the three lower frequency proposed stimuli, but for the intended relative presentations levels, as detailed in Table 1, when referenced to the ISTS at a level of 65 dB SPL. Further details are given in the Supplementary Material.

Figure 6 shows a comparison of the processed and averaged recordings from 100 clean examples of each stimulus. The top row shows the recordings for a middle-aged male participant, and the bottom row shows the corresponding recordings for the young female participant. The left-hand panels show the HEARLab recordings, the middle panels show the recordings of the proposed stimuli each presented at 65 dB SPL, and the right-hand panels show the recordings of the proposed stimuli at the correct relative levels “Relative SPL”, as detailed in Table 1. Despite the mild high-frequency loss in one ear of the male participant (max 30 dB HL), the waveforms are “textbook” for all stimuli from both sets, showing a distinct P1-N1-P2 complex, with P2 timed around 200 ms, and a high response level. For the female participant, the waveforms are smaller and noisier, but distinct. The low-frequency stimulus in each set generally shows a longer latency than the two higher frequency stimuli from each set.

All HEARLab-derived waveforms showed a significant detection of a synchronized deviation from the baseline response using the Hotelling *T*^2^ test, *p* < 1e-19 for the male participant, and *p* < 1e-6 for the female participant. Despite the much lower presentation levels for the mid and mid-high signal, clear responses have been evoked in both participants (right-hand panels). Similarly, all new-stimuli-derived waveforms show a significant detection at *p* < 1e-8, except for the mid-high stimulus in the young female, presented at speech-relative level, where *p* = .0021. The “relative level” stimuli, despite their intended, low, presentation levels did not fail to obtain a response.

## The Effects of Hearing Aid Processing on Short-Duration Stimuli

Hearing aid signal processing contains multiple stages of nonlinear processing and therefore can affect the spectrotemporal pattern of the stimulus and the consequent evoked response (Billings, Tremblay, Souza, & Binns, 2007). Apart from dynamic range compression, aids may incorporate dynamic range expansion at low input levels (Plyler, Trine, & Hill, 2009). Such expansion effectively switches off the aid and removes low-level noise, generated either internally or externally to the aid, which may cause irritation to the wearer. Associated with such expansion, as with dynamic range compression, are attack- and release-time constants. These effectively determine the rate at which the aid switches on and off. If the attack time is too long, it is therefore possible for a brief low-level signal to have its temporal envelope heavily distorted as the gain is increased at the onset of the signal. Jenstad, Marynewich, and Stapells (2012) reported on the effect of three unnamed hearing aids (two digital and one analog) on the processing of either short-duration (60 ms) or long-duration (757 ms) 1-kHz tone bursts, at three different input levels, 30, 50, or 70 dB SPL. Both digital aids distorted the temporal envelope of the 30 dB SPL stimuli, reducing their effective duration. For the longer duration stimuli at a presentation level of 30 dB SPL, there were also more subtle effects at the onsets, differing between aids. If distortion of the temporal envelope of short-duration stimuli is a regular occurrence in hearing aids and the gain applied by the hearing aid is wildly different from that intended by the insertion gain prescription formula, then the use of these types of stimuli to assess hearing aid performance is questionable.

Easwar, Purcell, and Scollie (2012) compared the insertion gains of ten hearing aids in response to each of eight phonemes presented either in isolation or in running speech. Their isolated phonemes were presented in a way similar to their use in measures of CAEP, short bursts with an interstimulus interval of 1,125 ms. They reported that the difference in aided level of phonemes in isolation compared to the aided level in running speech was typically in agreement for about 70% of the test conditions, but exceeded 3 dB for the remaining test conditions. Their worst case difference was around 8 dB. The direction of any difference was generally lower for the isolated phoneme, although there may have been an overshoot at phoneme onset that briefly increased the level relative to that found in running speech. Since phonemes are wideband stimuli, then, after amplification, their reported measures of overall level may miss subtleties that occur in narrow frequency ranges of the stimuli. Consideration of this effect is important, so we performed a similar set of measures with our more frequency-specific stimuli as well as in a wider range of presentation contexts.

**Figure 7.**
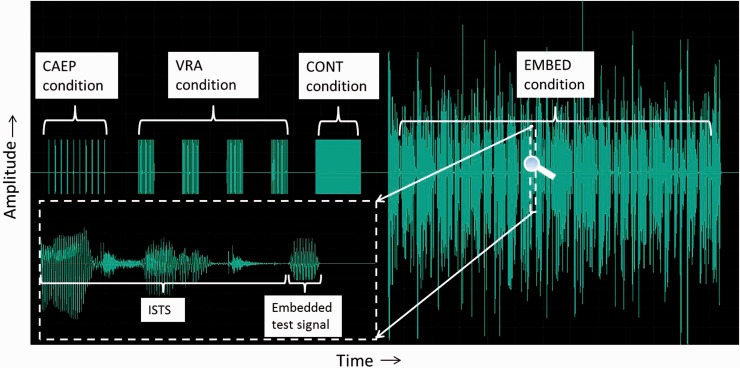
Structure of the test signal to assess hearing aid insertion gain responses to probe stimuli in four different contexts: (a) CAEP condition: one pulse per 1.1 s, as used in infant CAEP testing. (b) VRA condition: a block of 12 bursts of four pulses per second over 3 s, with a block being repeated 4 times, separate by gaps of 5 s. (c) CONT condition: concatenation of 100 pulses into a continuous burst. (d) Embedded condition: 22 separate pulses were inserted into temporal gaps of the ISTS signal, as exampled in the expanded portion of the waveform (inserted at left bottom).

To measure the variation of gain applied by a hearing aid in response to the presentation pattern of the proposed stimuli, a test signal was crafted consisting of four variations of sequences of the test stimuli used. Two of these sequences were intended to imitate conditions in which the stimuli were to be used, as well as two more theoretical conditions which were intended to probe aspects of the hearing aid signal processing. The time waveform of the test signal is shown in Figure 7. Each variant is separated from its neighbor by a period of five seconds of silence. The variants were as follows:

1. A CAEP-like condition consisting of 10 repetitions of the test signal at a rate of 0.9 Hz. This was the presentation rate used in a concurrent study on infant aided CAEPs being performed by author AV.
2. The Visual Reinforcement Audiometry (VRA) condition consisting of an initial block of 12 test signals, presented at a rate of 4 Hz. This faster rate has been used to attract infants’ attention for the purposes of behavioural testing (Van Dun et al., 2012). Three more blocks of 12 test signals at the VRA rate were presented with a five second silence in between each block. Each block was therefore three seconds long representing a typical presentation length for a VRA stimulus.
3. The continuous (CONT) condition consisting of 100 repetitions of the test signal with no gaps in between individual stimuli. Never intended as a presentation condition to real hearing aids, this condition was intended to explore likely adaptive behavior in the hearing aid signal processing in response to noise-like stimuli.
4. The EMBED condition, comprised 60 s of the ISTS stimulus with 22 examples of the test stimulus embedded in natural gaps in the speech pattern (see the expanded portion of Figure 7 for an example).

Test signals of identical format were generated separately for the low, mid, and mid-high stimuli. The level of the test bursts was set at the same relative level to the mean of the ISTS signal, as detailed in Table 1. The high-frequency stimuli was not tested since, at the time of testing, hearing aids capable of delivering bandwidths with high power were not generally available in the clinical population.

The same infant-oriented research project mentioned in (1) earlier provided four examples of clinically fitted behind-the-ear hearing aids programmed to alleviate a range of hearing losses in infants with ages less than 12 months. These aids were a Phonak Sky Q70SP, an Oticon Sensei Pro, a Phonak Nios, and an Oticon Mini synergy. A brief description of the essential features of each aid is given in separate rows of Table 2.

**Table 2.** Summary Details for Hearing Aids Used to Assess Insertion Gain Responses to Probe Stimuli in the Four Different Contexts (CAEP, VRA, CONT, and EMBED).

Model	No. channels	Attack time (ms)	Recovery time (ms)	Aid fitting range	Degree of loss fitted to	Features active in specific fitting
Phonak Nios S H20 V	16	10	50	Mild-to-severe	Mild-moderate	Soundflow
Phonak Sky Q70-SP	16	1	50	Mild-to-profound	Moderate	Soundflow, frequency compression
Oticon Sensei Pro BTE (90)	16	Depends on fitting	Depends on fitting	Mild-to-severe	Moderate	General pediatric program
Oticon Spirit Synergy MiniBTE (85)	16	Depends on fitting	Depends on fitting	Mild-to-severe	Mild	General padiatric program

*Note*. The sixth column indicates the degree of loss being compensated for by the specific aid used in the measurements.

The experimental method is detailed in the Supplementary Material. Basically, the response of each hearing aid to the stimuli presented in the free-field at 50, 65, and 80 dB SPL was recorded in the coupler of a manikin. Occluded delivery was used to reduce the effect of the external sound field adding to the hearing-aid processed sound. In addition to the hearing-aid recordings, an open-ear recording was also made in order to provide a reference for the calculation of insertion gains.

### Measurements

The recordings were analyzed using MATLAB™ to measure the RMS amplitude of the stimuli within each of the presentation conditions, across the middle 50 ms of each stimulus (i.e., avoiding onset and offset ramps). We did not observe any major alteration of temporal envelope duration as reported by Jenstad et al. (2012). Differences in the gain settings of the measurement pre-amp were accounted for in making the calculations. To reduce the effect of the recording noise on the measures, each recording was band-pass filtered with a linear phase filter with a gain of 0 dB across the central portion, centered on each stimulus and extending to half octave above and below the edges of the stimulus.

**Figure 8.**
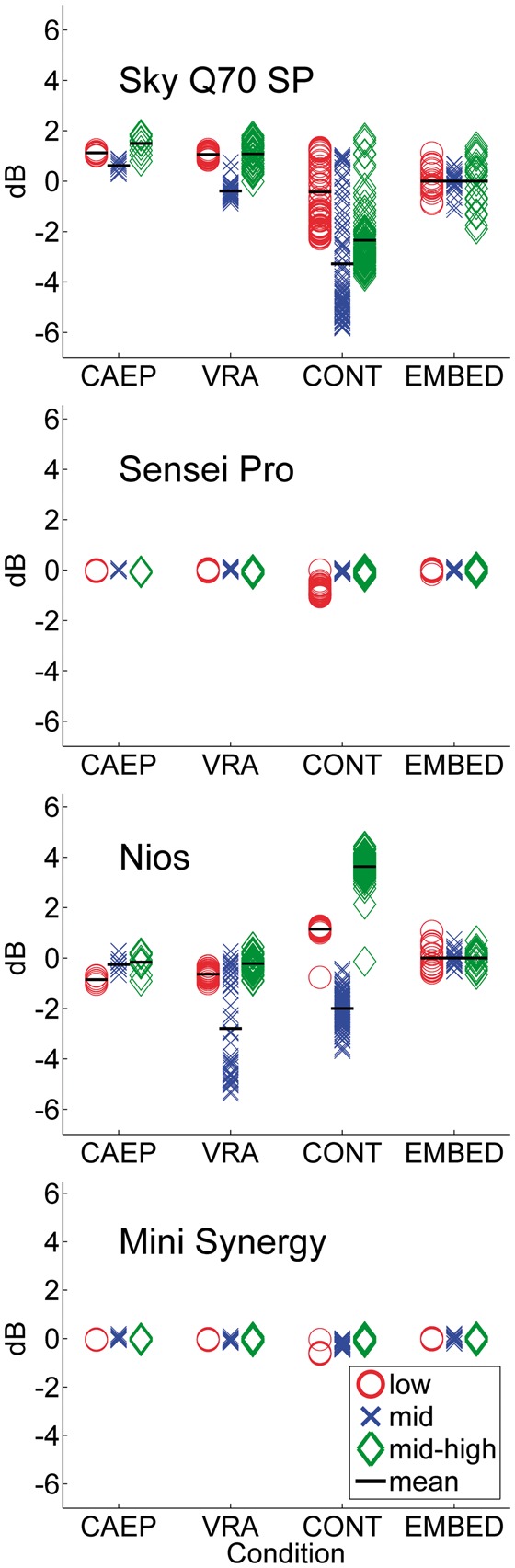
The insertion gains for each stimulus (separate symbols) and their mean (horizontal line) for each test condition and each hearing aid, referenced to the same stimulus in the EMBED condition with a speech presentation level of 65 dB SPL. The legend relates the symbol to its stimulus type. See text for further details.

Figure 8 shows the range of insertion gains for each pulse in each stimulus condition for the 65 dB SPL input level, referenced to the mean insertion gain achieved during the EMBED condition for the same stimulus type. The results for the 50 and 80 dB SPL input levels are reported and discussed in the Supplementary Material. The measured gains are shown on separate panels for each hearing aid and with separate symbols for each stimulus type as a function of the four variants of test signal condition, CAEP, VRA, CONT, and EMBED. Means are shown as black lines, but, for reasons of clarity, only for conditions where the scatter for individual pulses exceeds 1 dB.

For the Sensei Pro and the Mini synergy, there was very little variation in gain with change in the presentation loci of the test stimulus, for any of the stimulus types. For the other two aids, it was interesting to see that the gain for each stimulus type varied throughout the course of the continuous ISTS, presumably depending on the context of the speech local to the embedded pulse. One benchmark for assessing appropriate use of the stimuli for CAEPs and VRA would be that the variation seen in these two conditions was similar to, or less than that seen in running speech. This was true for all of the aids except the Nios when processing the mid-frequency stimulus in the VRA condition; we return to this shortly. Overall, the results showed smaller differences than reported by Easwar et al. (2012), and, for CAEP and VRA conditions, much closer to, and within the 3 dB range of “acceptable” difference assumed by Easwar et al. Without further recordings, we cannot be sure whether the discrepancy between their and our work is due to the increased frequency specificity of our stimuli or the lower number of hearing aids that we tested.

**Figure 9.**
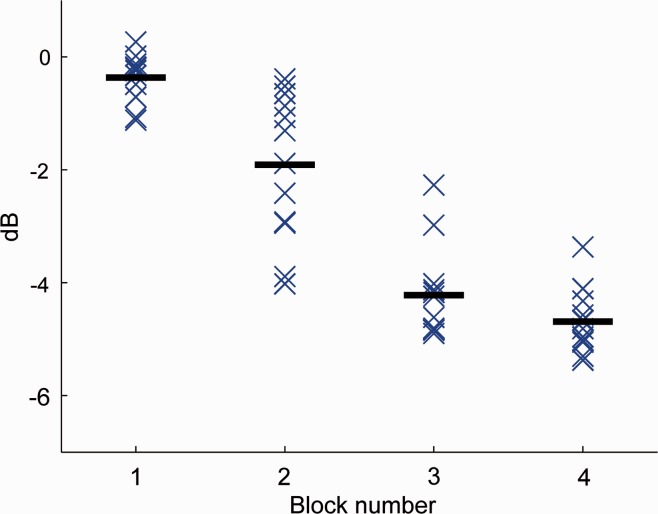
The relative insertion gain of the Nios aid to the mid-frequency stimulus in the VRA condition, separated by block number (time). The gain for individual stimuli is shown by crosses. Mean gain of each block is shown by a thick horizontal line. A progressive decrease in gain is seen with increasing block number, indicating some form of adaptation.

The Nios response to the VRA condition using the mid-frequency signal, where the mean gain difference was around 3 dB, but with a very wide range of individual levels, was examined further. This condition comprised four blocks of 12 stimuli, separated by 5 s. In Figure 9, the gain of each stimulus in a block was replotted, but separated by block number. The variation observed in the Nios was that of the gain successively decreasing during the course of each block (not shown), but also decreasing with increasing block number, indicating some form of adaptation. The difference between successive block means was 1.5, 2.3, and 0.5 dB. The differences between block means were all significant for comparisons between all blocks, except between Blocks 3 and 4 (*t* > 3.9, *df* = 22, *p* < .01, corrected for multiple comparisons). We are not privy to the time constants associated with this adaptation, but since pediatric VRA routinely involves waiting longer than 5 s to check for response, we suspect that this may be less of a problem. The mean gain in the initial block was only 1 dB lower than the average in the embedded condition. For the behavior shown, given the likely practical accuracy of the sound field in a clinical setting being within ± 3 dB, it was only by the third block that the stimulus would have been out of calibration. We have not yet investigated this further and is likely to vary both across and within different brands of aids, so this behavior remains as a caveat to the use of the stimuli in a VRA assessment. We suspect that longer interblock pauses, as are common in clinical VRA, would excite this behavior less, but such an investigation is beyond the scope of this article.

Adaptive gain behavior was also seen in the CONT version of the stimulus presentation, especially for the mid-frequency stimulus in both the Nios and Sky Q70 SP. This behavior was not unexpected since noise reduction had not been deactivated and the lack of speech modulation rates within the stimuli could be expected to excite the noise reduction feature.

Longer duration versions of the stimuli may be useful in the exploration of the ASSR (Picton, 2011), where the application of low-rate speech modulations (<32 Hz, Xu, Thompson, & Pfingst, 2005) while preserving the spectral constraint of the stimuli, should provide resilience against the adaptive behavior of noise-reduction processing found in digital hearing aids.

A similar pattern of results was observed for the same stimuli when presented at 50 and 80 dB SPL. The insertion gains as a function of input level for all four devices and three test stimuli are given in the Supplementary Material. Subtle variations from the results at 65 dB SPL are discussed in the same.

Overall, for both the CAEP and VRA conditions, apart from the long-term adaptive behavior of the Nios aid, there appear to be no major concerns as to the use of these stimuli in the CAEP and VRA conditions.

## Conclusions

A new set of four short-duration stimuli is proposed for the measurement of CAEP responses. Primarily designed for use in free-field presentation for validation of hearing aid fittings, the purpose of each stimulus is to produce a cochlear response that is relatively uniform across an integration bandwidth exceeding that found in impaired ears. The cochlear response for each stimulus is intended to be localized in both time and frequency so as to give greater precision as to the *what* and the *when* of the stimulus produced any measured CAEP responses.

The use of real-speech tokens for such a measurement purpose appears to contain potential confounds with defining the spectrotemporal locus of peak energy, the stimulus duration, the reference level for presentation, as well as the variability with change in physical acoustics such as the change in meatal length with age. Such confounds can be mitigated by judicious filtering, but the stimuli then lose their “speech” attributes.

By specifying the presentation level of each stimulus relative to the level of the ISTS, which is commonly used to verify hearing aid insertion gains, CAEP results are more transferable to assessment of audibility in the human ear. For clinical testing, an increase in presentation level over the theoretical level appears necessary in order to provide a minimum level of detectability of the CAEP within the waveforms.

Assessment through a sample of four modern digital hearing aids used in infant clinical fittings show that the signals survived processing with a level that was fairly independent of context of delivery conditions, except for adaptive gain applied to a multisecond duration continuous signal, for which the signals were not intended.

## Supplemental Material

TIA885568 Supplemetal Material1 - Supplemental material for A Set of Time-and-Frequency-Localized Short-Duration Speech-Like Stimuli for Assessing Hearing-Aid Performance via Cortical Auditory-Evoked Potentials Click here for additional data file. Supplemental material, TIA885568 Supplemetal Material1 for A Set of Time-and-Frequency-Localized Short-Duration Speech-Like Stimuli for Assessing Hearing-Aid Performance via Cortical Auditory-Evoked Potentials by Michael A. Stone, Anisa Visram, James M. Harte and Kevin J. Munro in Trends in Hearing

## Supplemental Material

TIA885568 Supplemetal Material2 - Supplemental material for A Set of Time-and-Frequency-Localized Short-Duration Speech-Like Stimuli for Assessing Hearing-Aid Performance via Cortical Auditory-Evoked Potentials Click here for additional data file. Supplemental material, TIA885568 Supplemetal Material2 for A Set of Time-and-Frequency-Localized Short-Duration Speech-Like Stimuli for Assessing Hearing-Aid Performance via Cortical Auditory-Evoked Potentials by Michael A. Stone, Anisa Visram, James M. Harte and Kevin J. Munro in Trends in Hearing

## Supplemental Material

TIA885568 Supplemetal Material3 - Supplemental material for A Set of Time-and-Frequency-Localized Short-Duration Speech-Like Stimuli for Assessing Hearing-Aid Performance via Cortical Auditory-Evoked Potentials Click here for additional data file. Supplemental material, TIA885568 Supplemetal Material3 for A Set of Time-and-Frequency-Localized Short-Duration Speech-Like Stimuli for Assessing Hearing-Aid Performance via Cortical Auditory-Evoked Potentials by Michael A. Stone, Anisa Visram, James M. Harte and Kevin J. Munro in Trends in Hearing

## References

[bibr1-2331216519885568] Agung K. Purdy S. C. McMahon C. M. Newall P. (2006). The use of Cortical Auditory Evoked Potentials to evaluate neural encoding of speech sounds in adults. Journal of the American Academy of Audiology, 17, 559–572. doi:10.3766/jaaa.17.8.3 16999251 10.3766/jaaa.17.8.3

[bibr2-2331216519885568] American National Standards Institute. (1997). ANSI S3.5-1997, American national standards methods for the calculation of the articulation index. New York, NY: Author.

[bibr3-2331216519885568] American National Standards Institute. (2007). ANSI S3.4-2007. Procedure for the computation of loudness of steady sounds. New York, NY: Author.

[bibr4-2331216519885568] Bardy F. Van Dun B. Dillon H. (2015). Bigger is better: Increasing cortical auditory response amplitude via stimulus spectral complexity. Ear and Hearing, 36, 677–687. doi:10.1097/AUD.0000000000000183 26039014 10.1097/AUD.0000000000000183

[bibr5-2331216519885568] Billings C. J. Tremblay K. L. Souza P. E. Binns M. A. (2007). Effects of hearing aid amplification and stimulus intensity on Cortical Auditory Evoked Potentials. Audiology and Neurotology, 12, 234–246. doi:10.1159/000101331 17389790 10.1159/000101331

[bibr6-2331216519885568] Brennan M. A. McCreery R. W. Kopun J. Hoover B. Alexander J. Lewis D. E. Stelmachowicz P. G. (2014). Paired comparisons of nonlinear frequency compression, extended bandwidth, and restricted bandwidth hearing-aid processing for children and adults with hearing loss. Journal of the American Academy of Audiology, 25, 983–998. doi:10.3766/jaaa.25.10.7 25514451 10.3766/jaaa.25.10.7 PMC4269381

[bibr7-2331216519885568] British Society of Audiology. (2016). *Recommended procedure for Cortical Auditory Evoked Potential (CAEP) testing* Retrieved from https://www.thebsa.org.uk/wp-content/uploads/2016/05/Cortical-ERA.pdf (free registration required).

[bibr8-2331216519885568] British Society of Audiology. (2018). *Guidance on the verification of hearing devices using probe microphone measurements* Retrieved from https://www.thebsa.org.uk/wp-content/uploads/2018/05/REMS-2018.pdf (free registration required).

[bibr9-2331216519885568] Burkard R. F. Don M. Eggermont J. J. (2006). Auditory evoked potentials: Basic principles and clinical applications. Philadelphia, PA: Lippincott Williams and Wilkins.

[bibr10-2331216519885568] Byrne D. Dillon H. Tran K. Arlinger S. Wilbraham K. Cox R. Kiessling J. (1994). An international comparison of long-term average speech spectra. The Journal of the Acoustical Society of America, 96, 2108–2120. doi:10.1121/1.410152

[bibr11-2331216519885568] Carter L. Golding M. Dillon H. Seymour J. (2010). The detection of infant Cortical Auditory Evoked Potentials (CAEPs) using statistical and visual detection techniques. Journal of the American Academy of Audiology, 21, 347–356. doi:10.3766/jaaa.21.5.6 20569668 10.3766/jaaa.21.5.6

[bibr12-2331216519885568] Cone B. Whittaker R. (2013). Dynamics of infant Cortical Auditory Evoked Potentials (CAEPs) for tone and speech tokens. International Journal of Pediatric Otorhinolaryngology, 77, 1162–1173. doi:10.1016/j.ijporl.2013.04.030 23722003 10.1016/j.ijporl.2013.04.030 PMC3700622

[bibr13-2331216519885568] Cone-Wesson B. Wunderlich J. (2003). Auditory evoked potentials from the cortex: Audiology applications. Current Opinion in Otolaryngology & Head and Neck Surgery, 11, 372–377. 14502069 10.1097/00020840-200310000-00011

[bibr14-2331216519885568] Easwar V. Purcell D. W. Scollie S. D. (2012). Electroacoustic comparison of hearing aid output of phonemes in running speech versus isolation: Implications for aided Cortical Auditory Evoked Potentials testing. International Journal of Otolaryngology, 2012, 518202. doi:10.1155/2012/518202 23316236 10.1155/2012/518202 PMC3536429

[bibr15-2331216519885568] Glasberg B. R. Moore B. C. J. (1990). Derivation of auditory filter shapes from notched-noise data. Hearing Research, 47, 103–138. doi:10.1016/0378-5955(90)90170-T 2228789 10.1016/0378-5955(90)90170-t

[bibr16-2331216519885568] Holube I. Fredelake S. Vlaming M. Kollmeier B. (2010). Development and analysis of an international speech test signal (ISTS). International Journal of Audiology, 49, 891–903. doi:10.3109/14992027.2010.506889 21070124 10.3109/14992027.2010.506889

[bibr17-2331216519885568] Hyde M. (1997). The N1 response and its applications. Audiology and Neurotology, 2, 281–307. doi:10.1159/000259253 9390837 10.1159/000259253

[bibr18-2331216519885568] Jenstad L. M. Marynewich S. Stapells D. R. (2012). Slow cortical potentials and amplification—Part II: Acoustic measures. International Journal of Otolaryngology, 2012, 386542. doi:10.1155/2012/386542 23193410 10.1155/2012/386542 PMC3502003

[bibr19-2331216519885568] Keefe D. H. Bulen J. C. Campbell S. L. Burns E. M. (1994). Pressure transfer function and absorption cross section from the diffuse field to the human infant ear canal. The Journal of the Acoustical Society of America, 95, 355–371. doi:10.1121/1.408380 8120247 10.1121/1.408380

[bibr20-2331216519885568] Keidser G. Dillon H. Carter L. O’Brien A. (2012). NAL-NL2 Empirical adjustments. Trends in Amplification, 16, 211–223. doi:10.1177/1084713812468511 23203416 10.1177/1084713812468511 PMC4040825

[bibr21-2331216519885568] Keidser G. Dillon H. Flax M. Ching T. Brewer S. (2011). The NAL-NL2 prescription procedure. Audiology Research, 1, 88–90. doi:10.4081/audiores.2011.e24 10.4081/audiores.2011.e24 PMC4627149 26557309

[bibr22-2331216519885568] Korczak P. A. Kurtzberg D. Stapells D. R. (2005). Effects of sensori-neural hearing loss and personal hearing aids on cortical event-related potential and behavioral measures of speech-sound processing. Ear and Hearing, 26, 165–185. 15809543 10.1097/00003446-200504000-00005

[bibr23-2331216519885568] Kuruvilla-Mathew A. Purdy S. C. Welch D. (2015). Cortical encoding of speech acoustics: Effects of noise and amplification. International Journal of Audiology, 54, 852–864. doi:10.3109/14992027.2015.1055838 26203722 10.3109/14992027.2015.1055838

[bibr24-2331216519885568] Lightfoot G. Kennedy V. (2006). Cortical electric response audiometry hearing threshold estimation: Accuracy, speed and the effects of stimulus presentation features. Ear and Hearing, 27(5), 443–456. doi:10.1097/01.aud.0000233902.53432.48 16957496 10.1097/01.aud.0000233902.53432.48

[bibr215-2331216519885568] Moore, B. C. J. (1995). *Perceptual Consequences of Cochlear Damage. *Oxford University Press, Oxford.

[bibr25-2331216519885568] Moore B. C. J. (2012). Effects of bandwidth, compression speed, and gain at high frequencies on preferences for amplified music. Trends in Hearing, 16, 159–172. doi:10.1177/1084713812465494 10.1177/1084713812465494 PMC4040859 23172008

[bibr26-2331216519885568] Moore B.C. J. Glasberg B. R. (2000). Frequency selectivity as a function of level and frequency measured with uniformly exciting notched noise. The Journal of the Acoustical Society of America, 108, 2318–2328. doi:10.1121/1.1315291 11108372 10.1121/1.1315291

[bibr27-2331216519885568] Moore B. C. J. Glasberg B. R. (2004). A revised model of loudness perception applied to cochlear hearing loss. Hearing Research, 188, 70–88. doi:10.1016/S0378-5955(03)00347-2 14759572 10.1016/S0378-5955(03)00347-2

[bibr28-2331216519885568] Moore B. C. J. Glasberg B. R. Baer T. (1997). A model for the prediction of thresholds, loudness and partial loudness. Journal of the Audio Engineering Society, 45, 224–240.

[bibr29-2331216519885568] Moore B. C. J. Glasberg B. R. Stone M. A. (2010). Development of a new method for deriving initial fittings for hearing aids with multi-channel compression: CAMEQ2-HF. International Journal of Audiology, 49, 216–227. doi:10.3109/14992020903296746 20151930 10.3109/14992020903296746

[bibr30-2331216519885568] Moore B. C. J. Stone M. A. Füllgrabe C. Glasberg B. R. Puria S. (2008). Spectro-temporal characteristics of speech at high frequencies, and the potential for restoration of audibility to people with mild-to-moderate hearing loss. Ear and Hearing, 29, 907–922. doi:10.1097/AUD.0b013e31818246f6 18685497 10.1097/AUD.0b013e31818246f6 PMC2688776

[bibr31-2331216519885568] Munro K. Purdy S. Ahmed S. Begum R. Dillon H. (2011). Obligatory cortical evoked potentials waveform detection and differentiation using a commercially available clinical system: HEARLab. Ear & Hearing, 32, 782–786. doi:10.1097/AUD.0b013e318220377e 21566525 10.1097/AUD.0b013e318220377e

[bibr32-2331216519885568] Patterson R. D. Robinson K. Holdsworth J. McKeown D. Zhang C. Allerhand M. (1992). Complex sounds and auditory images In Cazals Y. Demany L. Homer K. (Eds.), Auditory physiology and perception (pp. 429–446). Oxford, NY: Pergamon Press.

[bibr33-2331216519885568] Pearce W. Golding M. Dillon H. (2007). Cortical Auditory Evoked Potentials in the assessment of auditory neuropathy: Two case studies. Journal of the American Academy of Audiology, 18, 380–390. doi:10.3766/jaaa.18.5.3 17715648 10.3766/jaaa.18.5.3

[bibr34-2331216519885568] Picton T. W. (2011). Human auditory evoked potentials. San Diego, CA: Plural Publishing.

[bibr35-2331216519885568] Pittman A. L. (2008). Short-term word-learning rate in children with normal hearing and children with hearing loss in limited and extended high-frequency bandwidths. Journal of Speech, Language, and Hearing Research, 51, 785–797. doi:10.1044/1092-4388(2008/056) 10.1044/1092-4388(2008/056) PMC2529180 18506051

[bibr36-2331216519885568] Plyler P. N. Trine T. D. Hill A. B. (2009). The subjective evaluation of the expansion time constant in single-channel wide dynamic range compression hearing instruments. International Journal of Audiology, 45, 331–336. doi:10.1080/14992020600582224 10.1080/14992020600582224 16777779

[bibr37-2331216519885568] Rance G. Cone-Wesson B. Wunderlich J. Dowell R. C. (2002). Speech perception and cortical event related potentials in children with auditory neuropathy. Ear & Hearing, 23, 239–253. 12072616 10.1097/00003446-200206000-00008

[bibr38-2331216519885568] Seewald R. C. Moodie S. Scollie S. Bagatto M. (2005). The DSL method for pediatric hearing instrument fitting: Historical perspective and current issues. Trends in Amplification, 9, 145–157. doi:10.1177/108471380500900402 16424944 10.1177/108471380500900402 PMC4111493

[bibr39-2331216519885568] Stapells D. (2002). Cortical event-related potentials to auditory stimuli In Katz J. (Ed.), Handbook of clinical audiology (5th ed.) Baltimore, MD: Lippincott Williams and Williams.

[bibr40-2331216519885568] Stelmachowicz P. G. Pittman A. L. Hoover B. M. Lewis D. E. Moeller M. P. (2004). The importance of high-frequency audibility in the speech and language development of children with hearing loss. Archives of Otolaryngology–Head & Neck Surgery, 130(5), 556–562. doi:10.1001/archotol.130.5.556 15148176 10.1001/archotol.130.5.556

[bibr41-2331216519885568] Titze I. R. (1989). Physiologic and acoustic differences between male and female voices. The Journal of the Acoustical Society of America, 85, 1699–1707. doi:10.1121/1.397959 2708686 10.1121/1.397959

[bibr42-2331216519885568] Tremblay K. L. Billings C. J. Friesen L. M. Souza P. E. (2006). Neural representation of amplified speech sounds. Ear and Hearing, 27, 93–103. doi:10.1097/01.aud.0000202288.21315.bd 16518138 10.1097/01.aud.0000202288.21315.bd

[bibr43-2331216519885568] Tremblay K. L. Kalstein L. Billings C. Souza P. (2006). The neural representation of consonant-vowel transitions in adults who wear hearing aids. Trends in Amplification, 10, 155–62. doi:10.1177/1084713806292655 16959736 10.1177/1084713806292655 PMC4111424

[bibr44-2331216519885568] Van Dun B. Carter L. Dillon H. (2012). Sensitivity of Cortical Auditory Evoked Potential detection for hearing-impaired infants in response to short speech sounds. Audiology Research, 2, e13. doi:10.4081/audiores.2012.e13 26557328 10.4081/audiores.2012.e13 PMC4630953

[bibr45-2331216519885568] Wood S. A. Sutton G. J. Davis A. C. (2015). Performance and characteristics of the Newborn Hearing Screening Programme in England: The first seven years. International Journal of Audiology, 54, 353–358. doi:10.3109/14992027.2014.989548 25766652 10.3109/14992027.2014.989548 PMC4487563

[bibr46-2331216519885568] Wunderlich J. L. Cone-Wesson B. K. (2006). Maturation of CAEP in infants and children: A review. Hearing Research, 212, 212–223. doi:10.1016/j.heares.2005.11.008 16480841 10.1016/j.heares.2005.11.008

[bibr47-2331216519885568] Xu L. Thompson C. S. Pfingst B. E. (2005). Relative contributions of spectral and temporal cues for phoneme recognition. The Journal of the Acoustical Society of America, 117, 3255–3267. doi:10.1121/1.1886405 15957791 10.1121/1.1886405 PMC1414641

[bibr48-2331216519885568] Zhang V. W. Ching T. Y. C. Van Bunder P. Hou S. Flynn C. Burns L. McGhie K. Wong A. O. C. (2014). Aided cortical response, speech intelligibility, consonant perception and functional performance of young children using conventional amplification or nonlinear frequency compression. International Journal of Pediatric Otorhinolaryngology, 28, 1692–1700. doi:10.1016/j.ijporl.2014.07.022 10.1016/j.ijporl.2014.07.022 25128447

