# Supplementary figures and images for: A Set of Time-and-Frequency-Localized Short-Duration Speech-Like Stimuli for Assessing Hearing-Aid Performance via Cortical Auditory-Evoked Potentials
Source: Trends Hear. 2019 Dec 20;23:2331216519885568. doi: 10.1177/2331216519885568 (PMC6967206; doi:10.1177/2331216519885568)

80 dB input

Sky Q70 SP

Sensei Pro

Nios

Mini synergy

dB gain

65 dB input

50 dB input

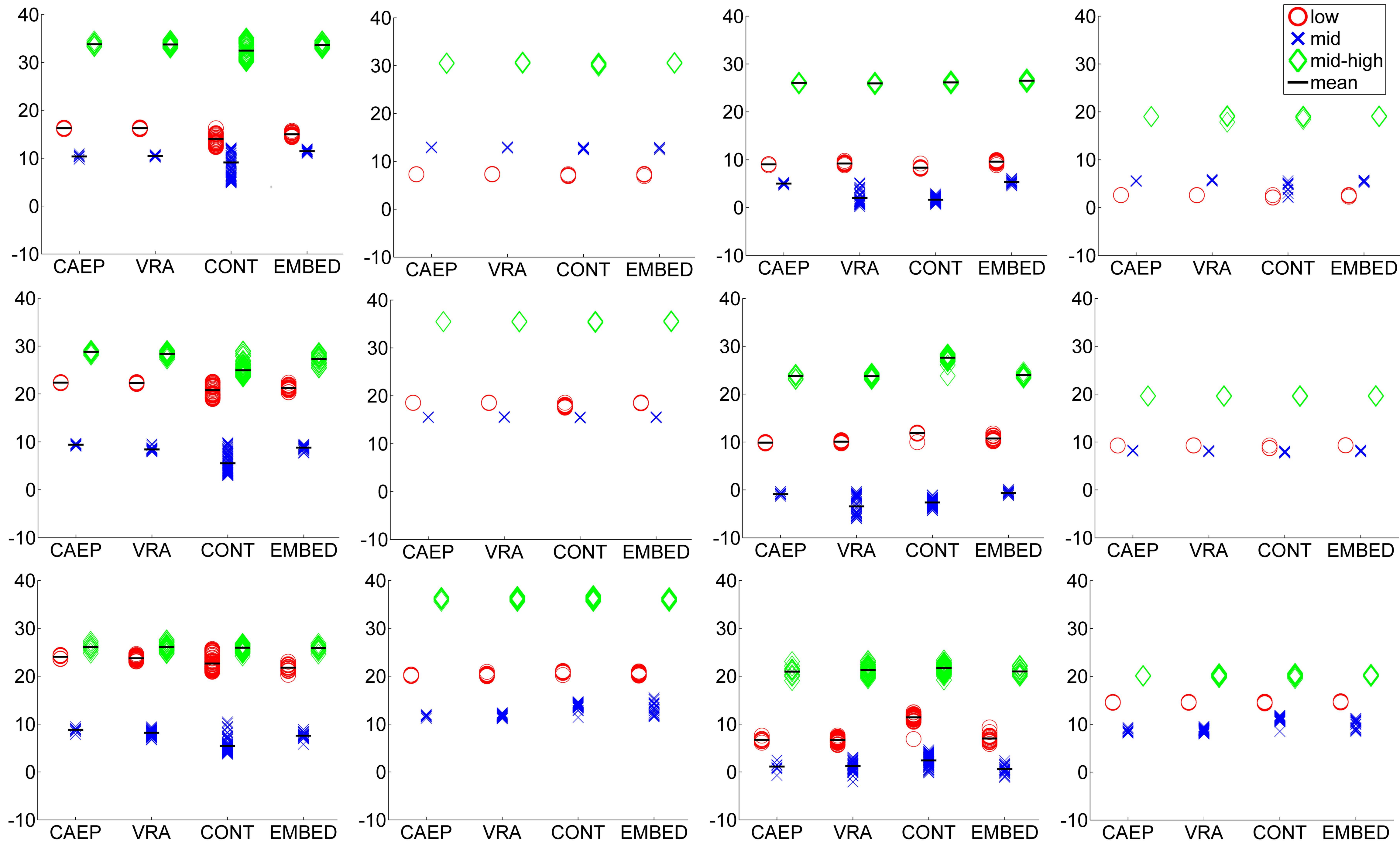

Condition

Supplement: TIA885568 Supplemetal Material1 - Supplemental material for A Set of Time-and-Frequency-Localized Short-Duration Speech-Like Stimuli for Assessing Hearing-Aid Performance via Cortical Auditory-Evoked Potentials [file TIA885568_Supplemetal_Material1.pdf]

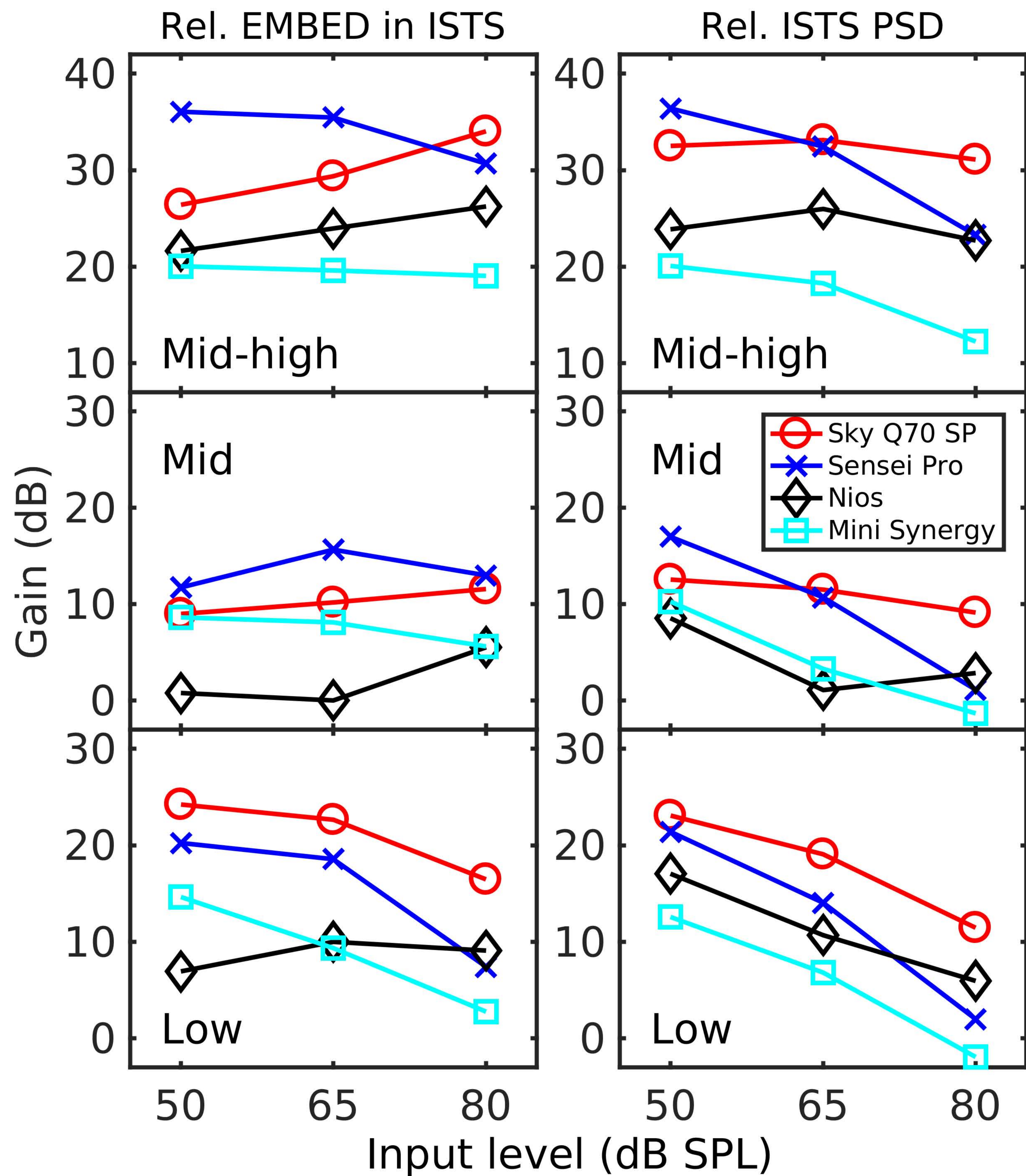

Supplement: TIA885568 Supplemetal Material2 - Supplemental material for A Set of Time-and-Frequency-Localized Short-Duration Speech-Like Stimuli for Assessing Hearing-Aid Performance via Cortical Auditory-Evoked Potentials [file TIA885568_Supplemetal_Material2.pdf]
