# Supplementary material for: A Set of Time-and-Frequency-Localized Short-Duration Speech-Like Stimuli for Assessing Hearing-Aid Performance via Cortical Auditory-Evoked Potentials
Source: Trends Hear. 2019 Dec 20;23:2331216519885568. doi: 10.1177/2331216519885568 (PMC6967206; doi:10.1177/2331216519885568)
Supplement: TIA885568 Supplemetal Material3 - Supplemental material for A Set of Time-and-Frequency-Localized Short-Duration Speech-Like Stimuli for Assessing Hearing-Aid Performance via Cortical Auditory-Evoked Potentials [file TIA885568_Supplemetal_Material3.pdf]

Supplement to:

A set of time-and-frequency-localised short-duration speech-like stimuli for assessing hearing-aid performance via Cortical Auditory-Evoked Potentials

Michael A. Stone[1,2], Anisa Visram[2], James M. Harte[3], Kevin J. Munro[2]

[2] Manchester Centre for Audiology and Deafness, School of Health Sciences, University of Manchester M13 9PL, UK

and

Manchester University Hospitals NHS Foundation Trust, Manchester, M13 9WL, UK

[3] Interacoustics Research Unit, Technical University of Denmark, Lyngby DK-2800, Denmark

[1] Corresponding author: michael.stone@manchester.ac.uk

Keywords

Cortical Auditory Evoked Potential, modulations, hearing aids, auditory perception

Experimental settings to measure CAEP responses from adults using either the HEARLab or the proposed stimuli

Recordings of evoked responses were performed in a sound-treated audiology booth of dimensions 4.9 x 3.5 x 2.5 metres (length x width x height). The stimuli were presented from a loudspeaker to a participant seated at 1-m distance. The axis between the participant and the loudspeaker was not parallel to either set of walls, in order to reduce the possible influence of reflections. CAEP recordings were made with either the HEARLab /m/, /g/ and /t/ stimuli presented using the HEARLab system with its accompanying single-cone loudspeaker, or the proposed low, mid and mid-high stimuli under control of an Eclipse system (Interacoustics, Middelfart, Denmark) For the latter system, a Tannoy VXP6 (manufactured by Tannoy Ltd, Coatbridge, Scotland) with dual-concentric cones was used for acoustic presentation.

All stimuli were presented binaurally, either all at the same level, 65 dB SPL, or, for one set of the proposed stimuli, presented at the intended relative levels (Table I of the main paper) referenced to the ISTS with an overall level of 65 dB SPL. The presentation levels for this last set were rounded to 62, 50 and 45 dB SPL for the low-, mid- and mid-high stimuli, respectively.

A three-electrode montage was used for recordings: left and right mastoid for ground and reference, and either vertex (middle-aged male participant) or high forehead (young female participant with clinically normal hearing) for signal. EEG recordings were stored on the respective control system, and the raw recordings exported for external processing. Thresholding for noise rejection (100 μVolts), as well as subsequent filtering (1 – 30 Hz) was set as near as possible the same between the two systems. 100 clean presentations per stimulus were obtained from each system, in blocks of 25 when using HEARLab, and in blocks of 20 when using the Eclipse. The three stimuli from each set were presented interleaved in the blocking order for the respective systems used to gather the data. The participants were awake during the recordings. In order to maintain attention and consequently better quality recordings, the female participant was watching a silent video with captioning for the dialogue. Traces of the processed data are shown in Fig. 6 of

the main paper.

Experimental settings to investigate the effects of hearing aid processing on short-duration stimuli

The hearing aids were sequentially attached to the right ear of a KEMAR™ head and torso simulator via standard earmould tubing and an ER3A eartip. Occluded delivery was used so as to reduce the effect of the external soundfield bypassing the hearing aid. In order to simulate a clinical session, the hearing aids were programmed as per the recorded clinical settings, with none of the adaptive features de-activated. The KEMAR was sited in the same sound-treated audiology booth as used for the recording of cortical responses reported earlier. The centre of KEMAR's head was 1 metre from, and perpendicular to, the front of the Tannoy VXP6,as described earlier). The KEMAR meatus was terminated by a IEC 711 coupler and a Bruel & Kjaer 4192 microphone. The microphone signal was conditioned by a Bruel & Kjaer 2669-L powered by a GRAS Type 12AA power module (incorporating a stepped-gain amplifier). The power module signal was fed to an Edirol UA-25 2-channel USB soundcard attached to a Linux PC running CoolEdit™ under Wine (a MicroSoft Windows™ emulator). The power module gain, of either +20 or +40 dB, was chosen so as to ensure use of the full electrical signal range of the soundcard without digital clipping. In addition to the hearing-aid recordings, an open-ear recording was made at the same presentation level in order to provide a reference from which insertion gains could be calculated. All recordings were made with a resolution of 16 bits.

Presentation levels from the free field were one of 50, 65 or 80 dB SPL, measured using a calibration signal but with the head and torso absent. This signal consisted of a continuous, long-duration, version of the mid-frequency stimulus, with the same rms amplitude as the 60-s duration ISTS used in the recording. A CEL-143 (Casella-CEL, Bedford, UK) sound level meter was used to set the level of the calibration track such that the calibration track was measured at the intended presentation level at the reference position, one meter in front of the speaker, without the KEMAR

in place.

The effects of hearing aid processing on short-duration stimuli at 50, 65 and 80 dB SPL

Figure S1 shows the insertion gains as a function of input level for all four devices and 3 test stimuli, under the same presentation conditions as for Fig. 8 of the main paper. Note the change in ordinate scale compared to Fig. 8 of the main paper. Within a particular device and across stimulus condition, the pattern of behaviour appears to be fairly consistent across level to that observed in Fig 8, namely that there is little variation in gain applied to individual stimulus bursts in the CAEP and VRA conditions, except for the VRA condition with the Nios aid.

However, looking at the gain variation applied to any particular stimulus, as a function of input level, which would typically be expected to be compressive, an expansive pattern was occasionally observed. To illustrate this better, data from Fig. S1 have been re-plotted as a function of input level in Figure S2. In separate panels, the left-hand column of Fig. S2 shows the mean gain applied to each of the three stimuli in the EMBED condition, as a function of input level, 50, 65 or 80 dB SPL, hence the column title 'Ref. EMBED in ISTS'.

(1) with the Sky Q70 SP expansive gain functions appear to be applied to the mid and mid-high stimuli while a compressive gain function was observed for the low stimulus.

(2) with the Nios, an expansive pattern appears to be applied to the mid and mid-high frequency stimuli. There is a possibility that some of these measures were confounded by hearing-aid produced noise, especially at the 50 dB SPL input level. An alternative source of confound would be for the low frequency stimulus, especially at low gains. The earplug used to deliver the sound into the KEMAR meatus may not have attenuated the direct sound sufficiently that it did not contribute to the sound level recorded in the meatus.

However, hearing aids routinely implement dynamic range compression with multiple configurations, possibly incorporating expansion over part of the dynamic range as well as different time constants in different stages. (Blesser, 1969; Souza, 2002; Blamey, 2005; Kates 2005;

Giannoulis et al. 2012). Since the internal configurations of hearing aids are usually proprietary to the manufacturer, the link between how a device performs on a standardised test signal compared to a real-world signal can be very difficult to predict. This is illustrated in the right-hand panels of Fig. S2, headed "Ref. ISTS PSD". These show the data for the gain responses for the same conditions as the left-hand panels, but measured by calculating the power spectral density of the ISTS signal in the EMBED condition, and summing the power within the same bandwidth as for the respective stimuli (detailed in Table I of the main paper). These gain functions generally show the more expected compressive behaviour as a function of input level. The previously observed expansive behaviour appears to be a by-product of the way that the dynamic range compression is implemented in the hearing aid: it appears to have a differential effect depending on the duration of the measurement window used to calculate the gain (60-70 ms compared to 60 s). It should be noted that, even within the bandwidth of a particular stimulus, the range of levels and relationship of frequency content co-varies with the duration of the window. The differential effect reflects a difference in signal statistics between those of our mean-level, steady-state stimuli, and the long-term mean level of a non-steady-state signal.

Despite this oddity, the main issue of this experiment is not majorly affected, namely that there is little variation in gain applied to individual stimulus bursts in the CAEP and VRA conditions, except for the adaptive behaviour observed in the VRA condition with the Nios aid. Hence we do not explore further this effect of input level.

References

Blamey P.J. (2005) Adaptive Dynamic Range Optimization (ADRO): a digital amplification strategy for hearing aids and cochlear implants. Trends Amplif 9, 77-98. https://doi.org/10.1177/108471380500900203

Blesser B.A. (1969) Audio dynamic range compression for minimum perceived distortion. IEEE Trans Audio Electro, 17:22-31. No DOI.

Giannoulis D., Massberg M., Reiss J.D. (2012) Digital Dynamic Range Compressor Design— A Tutorial and Analysis. J Audio Eng Soc 60, 399-408. http://www.aes.org/e-lib/browse.cfm?elib=16354

Kates J.M. (2005) Principles of dynamic-range compression. Trends Amplif, 9, 45-76. https://doi.org/10.1177/108471380500900202

Souza P.E. (2002) Effects of compression on speech acoustics, intelligibility, and sound quality. Trends Amplif 6, 131-165. DOI: 10.1177/108471380200600402

Table 1 : Frequency and relative level of sinusoidal components to make proposed CAEP stimuli to at least 16-bit precision.

**Low frequency : /m/**		**Mid frequency : /g/**		**Mid-high frequency : /t/**		**High frequency : /s/**	
Frequency (Hz)	Relative level (dB)	Frequency (Hz)	Relative level (dB)	Frequency (Hz)	Relative level (dB)	Frequency (Hz)	Relative level (dB)
140	-96.6	140	-89.3	2489.92	-115.2	6275.49	-111.5
280	-2.1	280	-94.8	2509.59	-113.1	6322.55	-109.3
420	-6.3	420	-99	2529.41	-109.5	6369.95	-106.0
560	-8.5	560	-101.2	2549.36	-104.6	6417.69	-101.4
700	-100.3	700	-102.9	2569.46	-98.5	6465.78	-95.8
840	-101.9	840	-104.6	2589.71	-91.5	6514.22	-89.2
980	-101.9	980	-105.8	2610.1	-83.7	6563	-81.9
		1120	-6.1	2630.64	-75.4	6612.14	-74.1
		1260	-6.4	2651.33	-66.8	6661.64	-65.9
		1400	-7.1	2672.17	-58.3	6711.49	-57.6
		1540	-7.6	2693.16	-50.0	6761.7	-49.4
		1680	-7.8	2714.3	-42.2	6812.28	-41.6
		1820	-58.4	2735.59	-35.2	6863.22	-34.3
		1960	-108.9	2757.04	-29.1	6914.53	-27.7
				2778.64	-24.2	6966.22	-22.1
				2800.4	-20.7	7018.27	-17.5
				2822.32	-18.5	7070.71	-14.1
				2844.39	-17.9	7123.52	-12.1
				2866.63	-18.0	7176.72	-11.3
				2889.02	-18.1	7230.29	-11.3
				2911.58	-18.3	7284.26	-11.3
				2934.3	-18.4	7338.62	-11.2
				2957.19	-18.5	7393.37	-11.2
				2980.24	-18.6	7448.52	-11.1
				3003.46	-18.7	7504.06	-11.1
				3026.84	-18.8	7560.01	-11.0
				3050.4	-18.8	7616.37	-11.0

				3074.12	-18.8	7673.13	-10.9
				3098.02	-18.9	7730.3	-10.9
				3122.09	-18.9	7787.88	-10.8
				3146.34	-18.9	7845.88	-10.8
				3170.76	-18.9	7904.31	-10.7
				3195.35	-18.9	7963.15	-10.7
				3220.13	-18.8	8022.42	-10.6
				3245.08	-18.8	8082.12	-10.6
				3270.21	-18.7	8142.25	-10.5
				3295.53	-18.7	8202.81	-10.5
				3321.03	-18.7	8263.82	-10.5
				3346.71	-18.6	8325.26	-10.5
				3372.58	-18.6	8387.15	-10.4
				3398.64	-18.5	8449.49	-10.4
				3424.88	-18.5	8512.28	-10.4
				3451.32	-18.5	8575.52	-10.3
				3477.94	-18.4	8639.22	-10.3
				3504.76	-18.4	8703.38	-10.2
				3531.78	-18.3	8768.01	-10.2
				3558.98	-18.3	8833.1	-10.2
				3586.39	-18.3	8898.66	-10.1
				3613.99	-18.2	8964.7	-10.1
				3641.8	-18.2	9031.22	-10.1
				3669.8	-18.1	9098.21	-10.1
				3698.01	-18.1	9165.7	-10.1
				3726.42	-18.0	9233.67	-10.1
				3755.03	-18.0	9302.13	-10.0
				3783.86	-18.0	9371.08	-10.0
				3812.89	-17.9	9440.54	-10.0
				3842.13	-17.9	9510.5	-10.0
				3871.59	-17.8	9580.96	-9.9
				3901.25	-17.8	9651.94	-9.9
				3931.13	-17.7	9723.43	-9.9
				3961.23	-17.7	9795.43	-9.8
				3991.55	-17.6	9867.96	-9.8
				4022.08	-17.6	9941.01	-9.8
				4052.84	-17.5	10014.59	-9.7
				4083.82	-17.4	10088.7	-9.5
				4115.02	-17.4	10163.35	-9.3
				4146.45	-17.3	10238.54	-9.1
				4178.1	-17.2	10314.27	-8.9
				4209.99	-17.1	10390.55	-8.7
				4242.1	-17.1	10467.38	-8.5
				4274.45	-17.0	10544.77	-8.3
				4307.03	-16.9	10622.72	-8.1
				4339.85	-16.8	10701.23	-7.9
				4372.9	-16.8	10780.31	-7.7
				4406.2	-16.7	10859.96	-7.5
				4439.73	-16.6	10940.19	-7.3
				4473.51	-17.2	11021	-7.1
				4507.53	-19.2	11102.39	-6.9
				4541.8	-22.4	11184.37	-6.7

				4576.31	-26.8	11266.95	-6.5
				4611.08	-32.4	11350.12	-6.3
				4646.1	-38.8	11433.9	-6.9
				4681.37	-46.0	11518.28	-9.0
				4716.89	-53.7	11603.27	-12.5
				4752.68	-61.8	11688.87	-17.3
				4788.72	-69.9	11775.1	-23.3
				4825.02	-78.0	11861.95	-30.2
				4861.59	-85.7	11949.43	-37.9
				4898.41	-92.9	12037.54	-46.1
				4935.51	-99.3	12126.29	-54.6
				4972.88	-104.8	12215.68	-63.1
				5010.51	-109.3	12305.71	-71.4
				5048.42	-112.4	12396.4	-79.1
				5086.6	-114.3	12487.75	-86.0
				5125.06	-114.8	12579.75	-91.9
						12672.42	-96.7
						12765.76	-100.1
						12859.78	-102.1
						12954.48	-102.7
						13049.86	-102.4
						13145.93	-102.2

Figure captions

Figure S1. Insertion gains of the four tested hearing aids as a function of input level (dB SPL), stimulus type and presentation condition. The legend relates the symbol to the stimulus type. Note the different ordinate scale range compared to that used in Fig. 8 of the main paper.

Figure S2. Insertion gains for the stimulus in the CAEP condition using the test signal shown in Fig. 8 of the main paper, as a function of the input level of the ISTS speech signal, 50, 65 or 80 dB SPL. The rows show the results for the low, mid and mid-high stimuli. The left hand column shows the results when the levels are calculated from the levels of the same stimulus measured in the EMBED condition. The right-hand column shows the results when the levels are calculated from the band power measured using the power spectral density (PSD) of the ISTS signal, summed over the bandwidth relevant for each stimulus. These bandwidths are detailed in Table I of the main paper.
